# Understanding clinical biochemistry through case-based concept mapping by the students

**DOI:** 10.1186/s12909-025-08373-3

**Published:** 2025-12-11

**Authors:** Yuh Ping Chong, Nikos Thomacos, Elizabeth Verghese, Ahsan Sethi

**Affiliations:** 1 Rural and Remote Health, College of Medicine and Public Health, Flinders University, Bedford Park, South Australia Australia; 2 School of Health and Biomedical Sciences, Royal Melbourne Institute of Technology (RMIT) University, Melbourne, VIC Australia; 3 Department of Public Health, College of Health Sciences, QU Health, Qatar University, Doha, Qatar

**Keywords:** Case-based learning, Clinical biochemistry, Concept mapping, Active learning

## Abstract

**Background:**

Contemporary clinical biochemistry practice involves responding to existing and new diagnostic challenges in real time. To better prepare future biochemistry graduates, we introduced real patient cases, which students used to develop concept maps. This study explores the impact of clinical case-based concept mapping by the students on their learning experience and academic outcomes.

**Methods:**

Participants were Master students undertaking clinical biochemistry in a laboratory medicine course at an Australian institution from February 2019 to June 2023 (*n* = 238). Students collaborated with peers to analyze case reports drawn from the provided literature resources and generated concept maps to elucidate the scientific rationale and biochemistry behind real-life diseases. Students completed an anonymous, prevalidated university-based online course questionnaire to evaluate their learning experience and the quality of teaching. Pre and post analysis of academic grades since the introduction of clinical case-based concept mapping was also undertaken. Descriptive and inferential statistics were calculated for the quantitative data, whereas a content analysis was conducted for the qualitative data.

**Results:**

A total of 98 out of 238 students responded to the course survey. A significant increase in students’ satisfaction following the implementation of clinical case-based concept mapping (*p* = .003) was found, with a concomitant improvement in their perception of teaching quality (*p* = .008). We also found a significant association of academic grades with clinical case-based concept mapping supported with higher grades and pass rates. Qualitative data indicated that students found that the use of patient case reports and concept maps enhanced their knowledge retention, application, and critical thinking abilities. Besides that, the students also experienced collaborative learning and fostered interpersonal communication skills.

**Conclusions:**

Clinical case-based concept mapping by the students improves their satisfaction, perception of teaching quality, and overall academic outcomes. The exercise enhances curiosity to investigate real-life diseases and develop collaborative learning, critical thinking and problem-solving skills. Future research should explore this instructional design across other disciplines and fields.

**Supplementary Information:**

The online version contains supplementary material available at 10.1186/s12909-025-08373-3.

## Introduction

Previous research into the delivery of biochemistry education suggests that students are overwhelmed by the large amounts of information they need to accurately retain and reproduce to pass their examinations [1]. This is partly due to the mode of delivery as biochemistry has been taught in a classical lecture-based manner, where teachers provide direct instructions to students who remain passive recipients of knowledge [2]. There has been increasing calls for integration of basic sciences including biochemistry with clinical sciences for improved knowledge retention, development of clinical reasoning, interdisciplinary thinking and deep learning [3]. Thus, skills such as problem-solving and critical thinking that support evidence-based decision making are cultivated in students, rather than rote learning to pass examinations [2].

The introduction of various constructivist teaching approaches such as case-based learning (CBL) and problem-based learning (PBL) represented a paradigm shift in the way educators deliver medical education [4]. PBL is open enquiry that emphasizes on the process of discovery to solve open-ended problems with limited guidance from the facilitators; CBL is guided inquiry with facilitators providing direction for structured small group discussion [5, 6]. Both approaches encourage inquiry-based learning where students learn using a series of case studies or problems modelled after real-world scenarios that facilitate their conceptual clinical skills and ability to work with others [7]. To effectively address the scenario-based problems inherent in case studies, students need to be equipped with adequate background knowledge that they can then apply conceptual reasoning to derive practical and timely solutions [8]. It was demonstrated that learning through cases or problems reflects improved students’ professional knowledge, verbal communication and teamwork skills [9, 10].

While case study focused activities have been adopted in biochemistry education [11], it remains challenging for educators to translate clinical case materials into resources that promote deep learning and stimulate interests in students. A further challenge of small group learning in CBL or PBL relates to its ineffectiveness in engaging all group members to equally contribute to group work, especially when students lack an interest in the learning content and underprepared [12]. To resolve issues associated with PBL- and CBL-based teaching, contemporary medical education has focused on engaging creative tools to enhance the critical thinking skills in students strengthened by collaborative learning. One example is the jigsaw cooperative learning strategy, where students become content-experts on different sections of a topic and in turn share the knowledge with their peers to co-construct consensus understanding [13].

Concept maps are figural representations that help learners to organize knowledge by linking a sequence of concepts through connecting lines to visualize the crosstalk between concepts while digesting complex information [14]. Concept mapping has been utilized to impart basic science knowledge. For example, undergraduate students undertaking a biology course were instructed to draw flow maps of physiological reflex pathways, the core concepts in physiology routinely practiced by medical or biomedical professionals [15]. Constructivism emphasizes that learning is best achieved when learners interpret meaning from new information by reflecting on their own experiences [16]. Consolidating basic knowledge through concept mapping involves integration of prior knowledge and new information, prepares students to adapt and be able to apply fundamentals to new situations, aligning with the pedagogy of constructivism [17]. Organizing information and linking it to a schema is an example of cognitivism – that is, the active mental processes that retrieve and integrate information from memory [18]. The current study sought to address the limitations of the conventional PBL and CBL models – students who are disengaged and unprepared may be reluctant to contribute knowledge proactively to group discussions. To address such a challenge, we introduced patient case studies from the literature, which were used by our students to develop concept maps in biochemistry. We asked the question “What are the effects on student learning, engagement, and outcomes with the introduction of concept maps combined with CBL?” Our approach highlights the use of real-life clinical cases sourced from peer-reviewed literature to drive curiosity to investigate. By applying their theoretical knowledge, students worked collaboratively in small groups to integrate patient history, symptom onsets, diagnostic test results, disease mechanisms (pathophysiology) and diagnosis from clinical cases, which were then summarized and presented in the format of concept maps. Drawing upon constructivism and extending the educational value of inquiry-grounded CBL/PBL, this study explores the impact of clinical case-based concept mapping by the students on their learning and academic outcomes in biochemistry.

## Methods

### Participants

The study was conducted at Royal Melbourne Institute of Technology (RMIT) University, Australia, from February 2019 to June 2023. Ethical approval for the study was obtained from RMIT University Human Research Ethics Committee (HREC 26924). All students studying clinical biochemistry in the Master of Laboratory Medicine degree during the 5-year study period were invited to participate in the study (*n* = 238). The Master students were post-graduate students who had already completed their undergraduate qualifications elsewhere. The Master of Laboratory Medicine at the institution is a 2-year program that offers medical laboratory science focusing on medical laboratory diagnostic testing. The clinical biochemistry course described in this paper is one of the main subjects undertaken by the students as part of their studies. Our research utilized secondary, deidentified data obtained through the University’s course experience survey. No participants were recruited thus informed consent to participate is not applicable in this instance.

### Clinical Case-Based concept map

**Fig. 1:**
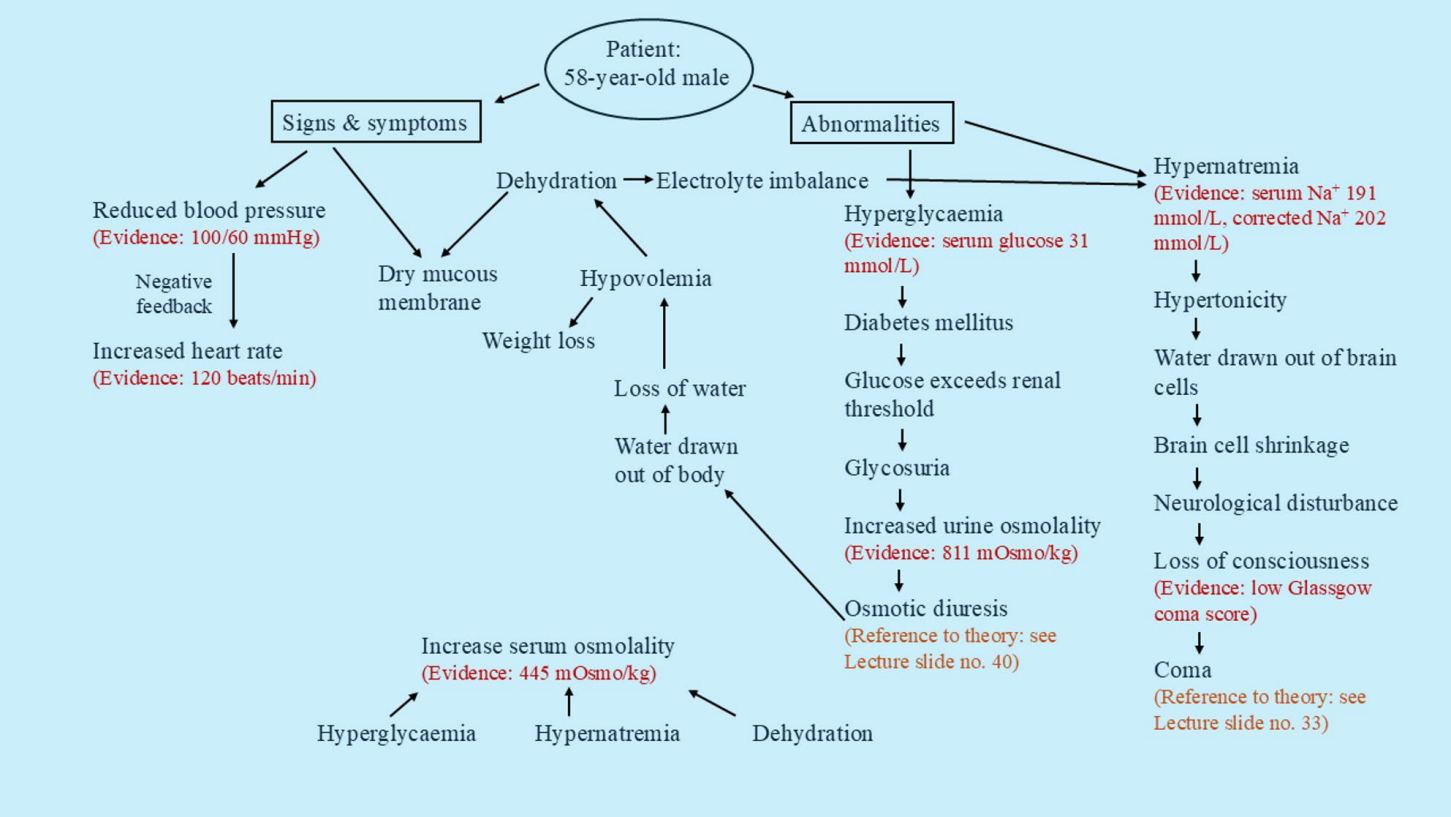
A real patient article that aligns the topic’s learning objective with questions derived from the article (upper panel) and an example concept map created by the students (lower panel)

Drawing on constructivism and cognitivism, we aimed to support our post-graduate students to problem-solve by applying knowledge using engaging and cognitively stimulating resources. The entire clinical biochemistry course was revamped. The individual modules across the teaching period can be viewed as supplemental information (see Additional file 1). In 2019, didactic lectures were delivered to all students on campus. Starting in 2020, classical lecture-style teaching was replaced with a student centered, active learning approach that paired concept maps with real patient case-based learning. Following a flipped classroom approach, lectures explaining the fundamental concepts were pre-recorded [19]. The pre-recorded lectures were broken down into shorter subtopics making the content easier for students to digest. Each subtopic, approximately 20 min long, covered up to 3 key concepts. These were uploaded to our institution’s learning management system. Students reviewed them at their own convenience before attending in-class sessions. The purpose of introducing pre-recorded lectures was to enhance independent and flexible learning, because students could access them at their own convenience. Each set of lectures conveyed concepts pertaining to the clinical biochemistry course’s learning objectives compulsory for students to undertake. Students’ participation in viewing the lectures was monitored by the analytics of the learning management system. To connect theory with real world clinical scenarios, journal articles with real patient cases that align with the course’s learning objectives were selected from the scientific literature by the first author, who was also the course coordinator. For example, an article by Xiao et al. described a real patient with extreme hyperglycaemia and hypernatremia compounded with severe dehydration [20]. Students applied knowledge gained from the pre-recorded lectures to synthesize clinical reasoning that linked hyperglycaemia, hypernatremia and dehydration. Topic-related questions centered around disease development were created based on the content of the article. One of these specifically asked students to construct concept map to explain the underlying cause of disease in the real patient and linked the concepts with theory covered in the lectures. During in-class sessions (2-hour tutorial per week), students collaborated with peers to construct flow diagrams on paper explaining the scientific basis contributing to the disease, a process known as concept mapping [21]. Students worked in small groups of approximately 6 students per group. Each group created a paper-based concept map, which was reviewed by a tutor (also the first author). The concept mapping technique encouraged students to apply theoretical knowledge gained from lectures to contextualize real-life disease development, while consolidating their clinical reasoning skills. The tutor provided feedback on all the concept maps developed to the whole class, with an emphasis on the reasoning behind disease development corroborated by theory from the lectures. An example of concept map generated by the students which has been digitally re-created is illustrated in Fig. 1. Of note, the tutor was the same in both pre- (2019) and post-implementation (2020–2023) periods, thus ensuring a consistent approach to course delivery throughout the study.

**Table:** 

Learning objective		Diagnosis and Investigation of Hypernatremia
Example questions derived from the article		o Describe the clinical use of corrected serum sodium in this case study. o Comment on the patient’s electrolytes and glucose levels post-admission​. o Predict the patient’s serum/urine osmolality. Provide your reasoning.​ o Describe the approaches taken to lower the patient’s serum sodium. o Draw concept map explaining the underlying cause of disease.​ Link your concepts with theory covered in the lectures.
Article/Reference		Xiao et al. Survival Following Extreme Hypernatremia Associated with Severe Dehydration and Undiagnosed Diabetes Mellitus. Case Rep Endocrinol; 2019:417 4259

### Course experience survey (CES)

The university has developed a prevalidated CES survey questions to capture students’ opinions on the quality of teaching (Mean Good Teaching Score – mGTS), together with their satisfaction with the course (Mean Overall Satisfaction Index – mOSI) [22]. Questions included in the survey are available in the supplementary data (see Additional file 2). A 5-point Likert scale was used; where 1 = strongly disagree, 2 = disagree, 3 = neutral, 4 = agree and 5 = strongly agree. A free-text option was available to provide qualitative feedback about the course. CES data and qualitative student feedback were collated by administrative staff and only deidentified data set was provided to the authors.

### Data collection

Data from 238 students enrolled in the Master of Laboratory Medicine clinical biochemistry course at RMIT University from February 2019 to June 2023 were used in the current study. All students who completed the course were invited to complete an online survey towards the end of a 12-week teaching semester to evaluate their experience of the course (CES). The student cohort in 2019 completed the course before implementation of the concept-map based approach (*n* = 64) were compared with the 2020–2023 cohorts, who were instructed using concept mapping (*n* = 174). All students in the course received an email that contained a link to the survey. Participation in the CES was voluntary, anonymous, and confidential.

### Student assessment of knowledge

Students’ knowledge was assessed throughout the semester using take-home assignments, multiple choice questions, and/or invigilated written theory examinations. The end-of-semester examination assessed students’ knowledge through their responses to scenario case-based questions. The marking criteria evaluated their reasoning process in explaining the causes of disease and underlying pathophysiology using concept map. Data regarding the student results were categorised into 5 subgroups – High Distinction (HD) = 80–100%, Distinction (DI) = 70–79%, Credit (CR) = 60–69%, Pass (PA) = 50–59% and Fail (NN) = 0–49%.

### Data analysis

The change in student evaluations of the course post intervention was analyzed. One-sample *t*-tests were performed to compare the 2019 mOSI and mGTS mean scores (pre-intervention) with the 2020, 2021, 2022, and 2023 mOSI and mGTS mean scores (post-intervention) data using SPSS.v.29. Significance was assessed using one-sample *t*-tests and chi-square tests of independence, with a *p*-value < 0.05 considered as statistically significant. For the chi-square tests, data in 2019 were compared against each year during the post-intervention phase, i.e. 2020, 2021, 2022 and 2023. Graphs of student academic results in grade category (HD, DI, CR, PA and NN) and the pass/fail rates over time were constructed using Microsoft Excel (version 16.0 Microsoft Corporation). A content analysis was conducted for all free-text comments provided by the students in the CES surveys regarding their detailed learning experiences and study outcomes. Two authors were involved in coding thematic analysis. Discrepancies were resolved by seeking independent opinions from a third author.

## Results

**Fig. 2:**
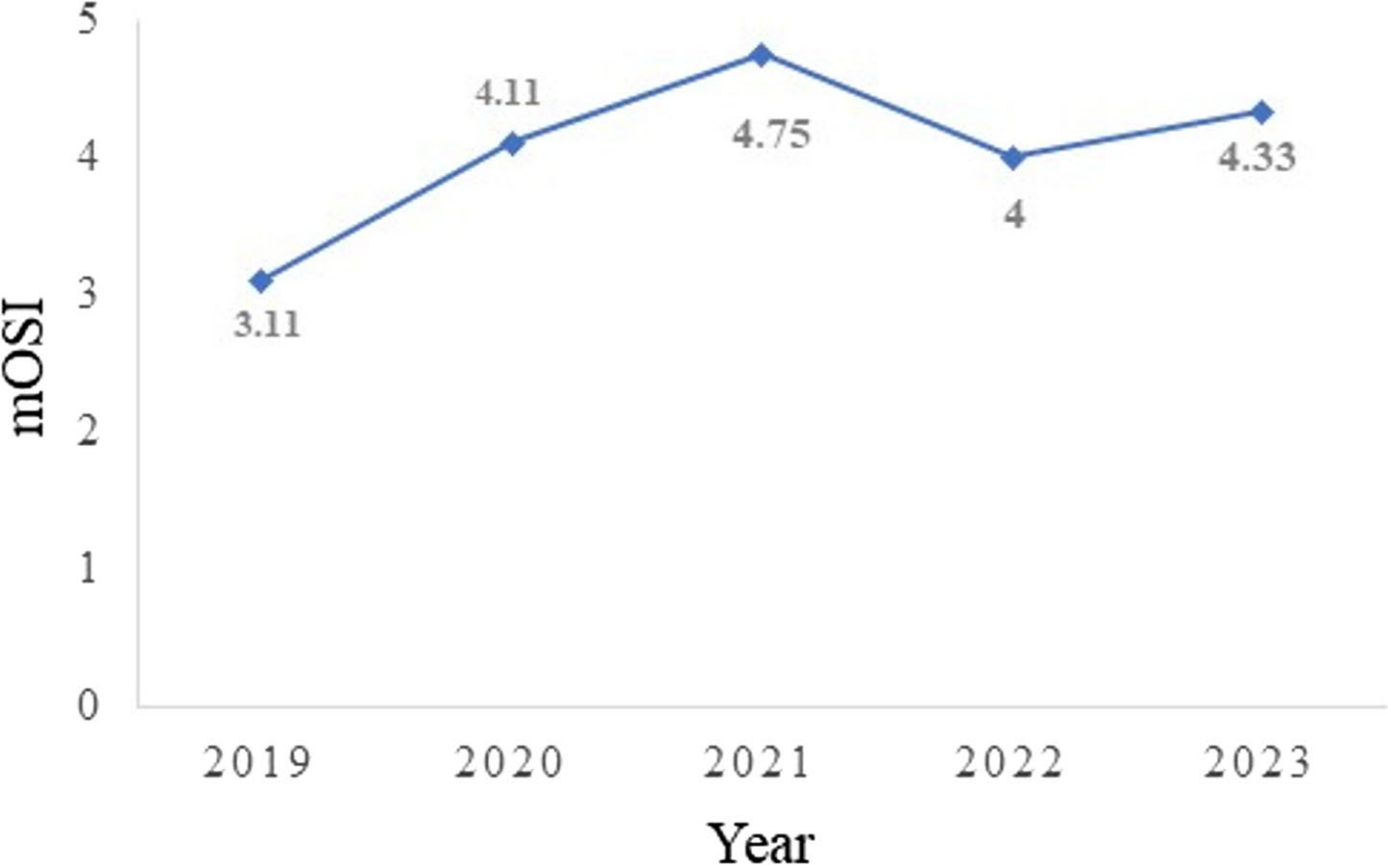
Mean Overall Satisfaction Index (mOSI) change over time before (2019) and after (2020–2023) the implementation of clinical case-based concept map in biochemistry teaching

**Fig. 3:**
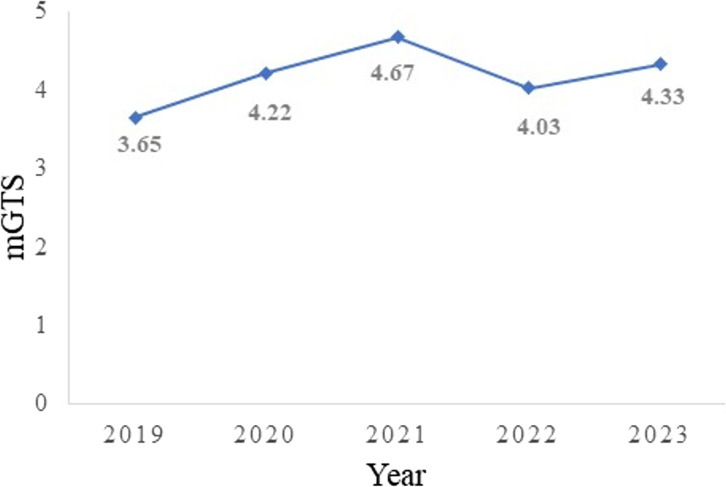
Mean Good Teaching Score (mGTS) change over time before (2019) and after (2020–2023) the implementation of clinical case-based concept map in biochemistry teaching

Of the 238 students who were enrolled, 65.5% were female and 34.5% were male. A total of 98 students completed the CES, which equates to 41% of the entire study population. A significant difference was found between the pre-intervention mOSI mean score (M = 3.11) and the post-intervention mOSI mean score (M = 4.30; SD = 0.33), [*t*(3) = 7.17, *p* =.003]. A significant difference between the pre-intervention mGTS mean score (M = 3.7) and the post-intervention mGTS mean score (i.e., teaching quality), was also found (M = 4.35; SD = 0.28) [*t*(3) = 4.97, *p* =.008]. Figures 2 and 3 illustrate the change over time in mOSI and mGTS scores since the introduction of clinical case-based concept mapping, respectively. Of note, a decrease in mOSI and mGTS scores was observed in 2022 relative to 2021, which may be explained by students’ post-pandemic transitioning from online to face-to-face learning, as well as their adaptation to the format of invigilated, closed-book assessments.

**Fig. 4:**
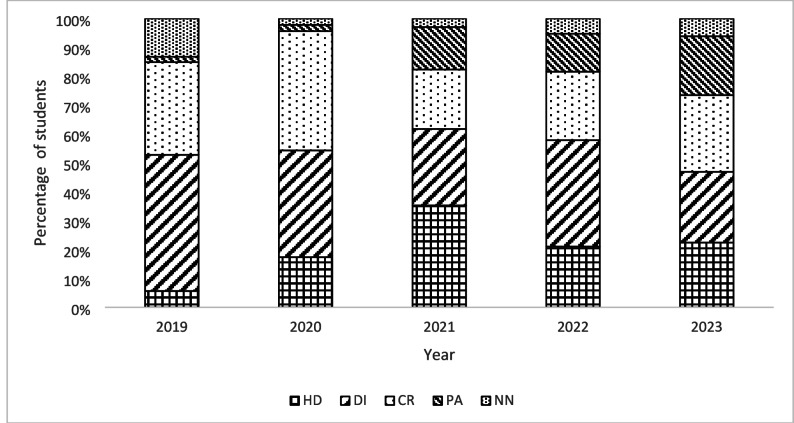
Student grade distributions before (2019) and after (2020–2023) the implementation of clinical case-based concept map in biochemistry teaching

Note: High Distinction (HD) = 80–100%, Distinction (DI) = 70–79%, Credit (CR) = 60–69%, Pass (PA) = 50–59% and Fail (NN) = 0–49%.

**Fig. 5:**
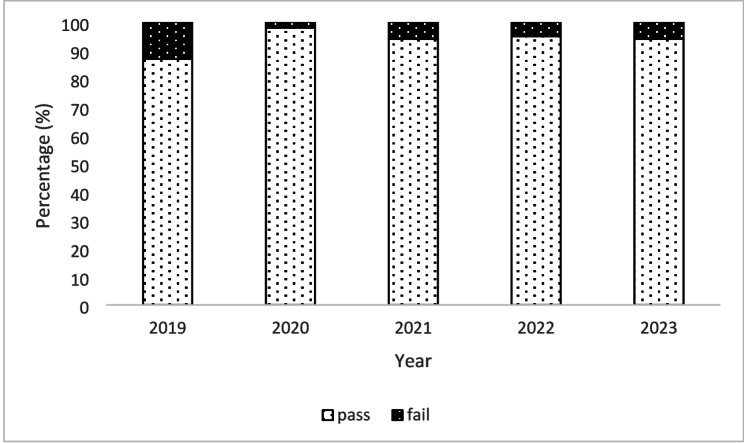
Percentage of students passing/failing before (2019) and after (2020–2023) the implementation of clinical case-based concept map in biochemistry teaching

We also examined the change in student grades over time since the introduction of clinical case-based concept mapping by analyzing the number of students in each grade category (HD, DI, CR, PA and NN). A chi-square test of independence showed that there was a significant association between grades achieved and clinical case-based concept mapping, *X*^2^ (4, *N* = 220) = 37.10, *p* =.002, with higher grades being achieved post-intervention. This was also the case regarding the number of students passing the course, *X*^2^ (4, *N* = 500) = 10.88, *p* =.028, with more students passing the course post-intervention. Figures 4 and 5 summarize these data. The pre-intervention pass rate in 2019 was 87%, meaning 13% of the students failed. Since the implementation of clinical case-based concept mapping, the fail rate has been reduced to less than 7% across four years from 2020 to 2023.

Data of the qualitative study are presented in Table 1. In analyzing the free-text comments provided by the students, several themes reflecting positive impact on knowledge retention, application and critical thinking abilities were identified. It appears that student learning was engaged through real patient case-based concept mapping that connects clinical reasoning to real-life scenarios, enhancing their critical thinking in the learning topics.

**Table 1:** Themes identified and example student comments

Theme	Student comment
Knowledge retention	I had the deepest understanding of the learning topic through CBL. It helped with retention of key ideas by repeating and applying in lectures and then tutorials. I liked the summaries at the end of each lecture and concept maps were very helpful in understanding theory.
Connect theory to practice	Case-study practice relates to real-world scenarios. Case studies were helpful in applying our knowledge. I felt that the knowledge and the skills that I acquired will undoubtedly help me when I enter industry. I found that the case study analysis really helped with bringing all the things learnt in the lectures together and made for a deeper understanding of the topic as well as a real-life approach. It helps me to understand what I might do in real world after completing the course. That means I am versed well with the theory as well as practical aspects. Concept maps - a crucial role in reinforcing what I learned during lectures. They provided an excellent opportunity to apply theoretical knowledge to real-life scenarios.
Critical thinking	Case studies with flow charts were a good opportunity to integrate our knowledge and challenge our analytical skills. The tutorial sessions with case study discussion were very helpful in practising theory and enhanced problem-solving skills. The highlight of this course, I think cases can help us improve critical thinking and understand the topic easily. I liked the way that several case studies were given for each week’s lecture, the discussion helped me develop critical thinking skills.
Collaborative learning	Tutorials were also a good way of applying our knowledge and working through problems together as a group.
Increased engagement	The contents were easy to follow and the concepts covered were very interesting. Articles were all interesting and complemented the weekly topics well. Overall, I am glad, I chose this major. I really liked all the case studies that expose us to the possible scenarios. Cases are good attempt. What I learnt in undergraduate period is all theory relative knowledge, which is dull, and I never have sense of achievement, but adding cases of different diseases gives me interest to investigate.

## Discussion

The current study presents an innovative approach to teaching clinical biochemistry using CBL alongside concept mapping that draws on real patient scenarios. Following the implementation of this approach, students’ satisfaction (*p* =.003) and their perception of teaching quality (*p* =.008) were significantly higher, and higher grades and pass rates were also achieved. Student feedback supports that real patient cases offered them an authentic learning experience, and the knowledge gained was relevant to their future practice. Furthermore, CBL plus concept mapping technique was found to be effective in students retaining knowledge and strengthening their critical thinking skills.

Students who are underprepared and disengaged in learning may continue to remain passive in CBL/PBL [12]. The real patient articles employed in our innovative teaching method aroused curiosity to explore the basic science underlying the complexity of real-life diseases, motivating students to learn through investigation. This led to a positive impact on the overall satisfaction of the clinical biochemistry course. To illustrate, an example article describing a patient with hypernatremia and hyperglycaemia [20] was used as a medium linking theory-based lectures to an actual scenario of how disturbance of body’s homeostasis contributed to disease states. Our students were enthusiastic in their investigations of how basic science can be applied to elucidate real-life diseases. This is consistent with a previous study that found CBL based on real patient narratives in a medical curriculum increased student engagement and promoted the application of scientific reasoning [23]. To further consolidate an inquiry and collaborative approach, we encouraged our students to develop concept maps in small groups to summarize the case-study using information presented in the article (e.g. patient’s history, patient’s diagnostic test results and differential diagnosis). Concept maps guided students in structuring evidence-based reasoning, connecting theory with practice which may lead them to problem-solve real world situations in their future careers. Of note, our clinical case-based concept mapping approach is not a worked example that provides a step-by-step solution to a problem or task to promote learning [24]. While the articles describe real-life diseases, they do not provide direct solutions to the concept maps. Rather, students must apply their background knowledge to correlate patient results and consider clinical scenarios presented in the articles to build logical concept maps. A benefit of using published case articles is that they are peer-reviewed and thus part of the scientific literature. Educators can freely access reliable and up-to-date clinical cases when designing learning materials; thus both cost-effective and time-efficient compared to creating clinical cases from scratch.

The process of constructing concept maps helped our students visualize relationships and sequences inherent in the case studies, and to critically evaluate whether their conclusions were sound and appropriate. Constructivist Learning Theory suggests that learners construct knowledge through exploration and by connecting new ideas with what they already know [17]. As such, concept mapping, an active learning strategy, allowed our students to synthesize new knowledge by linking concepts to existing knowledge, thus reinforcing and extending prior learning and clinical reasoning. Therefore, we feel that concept mapping based on real world clinical case studies scaffolds the development of higher order thinking and a critical, problem-solving mindset. A study in nursing education supports this claim, reporting that concept mapping enhanced critical thinking and facilitated nursing students successfully and appropriately planning and undertaking surgical procedures [25]. Further support for this claim comes from a study of a biology course, which demonstrated that the cognitive process of connecting ideas from multiple resources by creating a concept map resulted in deep rather than superficial learning [26].

Our approach also fostered new knowledge creation among students in a collaborative and team-based environment, as reflected in the student feedback. While we observed soft skill development as part of our study, it is important to note that such skills were not specifically evaluated. However, the literature suggests that collaborative learning fosters soft skill development (e.g. metacognition, communication skills, etc.). Metacognition refers to ‘thinking about thinking’, that is, an awareness of one’s own cognitive processes and the ability to control and adjust them to enhance learning and problem-solving [27]. As our students analyzed case articles in small groups to generate concept maps, this required collaboration to achieve a common goal, with students needing to reflect on their own and peer perspectives as part of the process. In other words, the co-construction of knowledge involved discussion, negotiation, and reflection to achieve a shared outcome. Looking forward to when students enter the workforce, effective communication and negotiation skills are critical when interacting with colleagues in the workplace [28]. Research conducted at Beijing University, involving 111 students, examined group performance in knowledge construction via computer-supported collaborative learning supports such a view [29]. Compared to the control group, students who participated in collaborative learning displayed a significantly greater capacity to synthesize new knowledge, as communication and negotiation among students facilitated team planning and goal setting.

## Limitations

The current study has several limitations: the design due to the nature of data available was cross-sectional. Since data were collected at a single point of time across the 5-year study period, we were unable to track behavioral changes of the same individuals over time. It remains to be determined how knowledge and soft skills gained through clinical case-based concept mapping can support our graduates in solving real world problems in their actual workplace. All students who completed the Master of Laboratory Medicine clinical biochemistry course from 2019 to 2023 were invited to complete the Course Experience Survey. The response rate was 41%, and the participation was voluntary. Consequently, the findings are not generalizable as our cohorts may not be representative of the larger population.

## Future directions

Future research would benefit from larger samples from different student cohorts to evaluate the impact of case-supported concept mapping technique in other disciplines and fields. Additionally, longitudinal studies from the classroom to practice are also suggested, given that such studies could investigate graduates’ ability to translate knowledge and skills attained when studying into their future professional practice.

## Conclusion

Clinical case-based concept mapping by the students improved their satisfaction, perception of teaching quality, and overall academic outcomes. The exercise enhanced curiosity to investigate real-life diseases and developed collaborative learning, critical thinking and problem-solving skills. Clinical cases sourced from scientific literature resulted in engaged learning, while concept maps facilitated the critical thinking and application of basic science knowledge to clinical context. This approach successfully bridged the gap between theory and contemporary biochemistry practice.

## Supplementary Information

Supplementary Material 1

## Data Availability

The datasets used and/or analyzed during the current study are available from the corresponding author on reasonable request.
