# Supplementary material for: Understanding clinical biochemistry through case-based concept mapping by the students
Source: BMC Med Educ. 2025 Dec 11;26:78. doi: 10.1186/s12909-025-08373-3 (PMC12801816; doi:10.1186/s12909-025-08373-3)
Supplement: Supplementary file 1 — Supplementary Material 1 [file 12909_2025_8373_MOESM1_ESM.pdf]

Additional file 1

Chong et. al. Understanding clinical biochemistry through case-based concept mapping by the students

The individual clinical biochemistry modules over the 12-week teaching period

Week	Module
1 – 3	*Laboratory management*<br>º Pre-analytical, analytical and post-analytical phases of medical laboratory diagnostic testing
4 – 12	*Core clinical biochemistry*<br>º Sodium and water<br>º Potassium<br>º Acid-base regulation<br>º Renal function<br>º Diabetes mellitus and hypoglycaemia<br>º Lipids and cardiovascular disease<br>º Endocrinology<br>º Calcium<br>º Liver function and bilirubin

Additional file 2

Chong et. al. Understanding clinical biochemistry through case-based concept mapping by the students

The course experience survey questions evaluating students' opinions on the quality of teaching (Mean Good Teaching Score – mGTS) and overall satisfaction (Mean Overall Satisfaction Index – mOSI)

Course experience survey (CES) items:

Quality of teaching (mGTS)

- The teaching staff are extremely good at explaining things.
- The teaching staff normally give me helpful feedback on how I am going in this course.
- The teaching staff in this course motivate me to do my best work.
- The teaching staff work hard to make this course interesting.
- The staff make a real effort to understand difficulties I might be having with my work.
- The staff put a lot of time into commenting on my work.

Student satisfaction (mOSI)

- Overall, I am satisfied with the quality of this course.
